# Tumor–Immune Cell Crosstalk Drives Immune Cell Reprogramming Towards a Pro-Tumor Proliferative State Involving STAT3 Activation

**DOI:** 10.3390/cancers18010116

**Published:** 2025-12-30

**Authors:** Karen Norek, Jacob Kennard, Kenneth Fuh, Robert D. Shepherd, Kristina D. Rinker, Olesya A. Kharenko

**Affiliations:** 1 Syantra Inc., 32 Royal Vista Drive NW, Suite 105, Calgary, AB T3R 0H9, Canada; karen.norek@syantra.com (K.N.); jacob.kennard@syantra.com (J.K.); kenneth.fuh@syantra.com (K.F.); bob.shepherd@syantra.com (R.D.S.); kdrinker@ucalgary.ca (K.D.R.); 2 Department of Biomedical Engineering, University of Calgary, 2500 University Dr NW, Calgary, AB T2N 1N4, Canada; 3 Department of Physiology and Pharmacology, University of Calgary, 3300 University Dr NW, Calgary, AB T2N 1N4, Canada

**Keywords:** tumor microenvironment, immune reprogramming, STAT3 inhibition, cancer-immune crosstalk, liquid biopsy, RNAseq, biomarkers, tumor-education, THP1, co-culture, breast cancer, TNBC, human monocytes

## Abstract

**Background/Objectives**: Tumor-induced immune reprogramming is increasingly recognized as a key mechanism by which cancers evade surveillance and promote disease progression. The interaction between cancer and immune cells within the tumor microenvironment (TME) can drive phenotypic and functional changes in immune populations, facilitating metastasis and immune evasion. **Methods**: In this study, we used co-culture models to expose THP1 monocytes to triple-negative breast cancer (TNBC) cells, MDA-MB-231 and BT-549, either directly or indirectly via tumor-conditioned media, to mimic tumor–immune cell communication. Transcriptomic and pathway analyses revealed that cancer-exposed monocytes adopt a reprogrammed phenotype marked by activation of pro-tumorigenic signaling pathways, enhanced proliferative capacity, and elevated expression of pro-inflammatory cytokines such as IL6. **Results**: Functional assays confirmed a significant increase in monocyte proliferation under both direct and indirect tumor exposure. Importantly, we demonstrated that this tumor-driven proliferation of THP1 cells could be suppressed by the STAT3 inhibitor STAT3-IN-12. This highlights the critical role of STAT3 signaling in mediating immune cell transformation and supporting a novel immunomodulatory approach for therapeutic intervention. **Conclusions**: These findings support the potential for targeting tumor-educated transcriptional programs as a novel immunomodulatory strategy in cancer treatment. Restoring immune cell homeostasis and suppressing pro-tumor phenotypes through pharmacological inhibition of the key signaling nodes such as STAT3 may complement existing cancer therapies. This study provides new insights into immune cell plasticity in cancer and identifies actionable strategies to counteract tumor-driven immune dysregulation.

## Simple Summary

Cancer can alter the behavior of immune cells to help tumors grow and spread. In this study, we explored how triple-negative breast cancer cells influence immune cells through direct contact and signals released by the tumor. We found that immune cells exposed to cancer cells become reprogrammed, showing increased growth and activation of pathways that support tumor progression. These changes were linked to a key signaling protein called STAT3. When STAT3 was blocked, the abnormal growth of monocytes was reduced, suggesting that targeting this pathway could help restore normal immune function. Our findings highlight how tumors manipulate immune cells towards a more pro-oncogenic phenotype and point to new strategies for improving cancer treatment by preventing immune cells from supporting tumor growth.

## 1. Introduction

Triple-negative breast cancer (TNBC) is an aggressive and heterogeneous subtype of breast cancer characterized by the absence of estrogen receptor, progesterone receptor, and HER2 (Human epidermal growth factor receptor 2) expression. This subtype is associated with a higher propensity for metastasis and significantly contributes to breast cancer-related mortality. The tumor microenvironment (TME) plays a pivotal role in facilitating malignant progression through complex cell–cell interactions that can modulate the landscape and function of immune cells [1].

While immune cells are inherently responsible for surveillance and elimination of transformed cells, tumors have evolved to reprogram immune cells into tumor-promoting phenotypes, a process often referred to as tumor education. This reprogramming leads to the loss of immunosurveillance, a critical event in breast cancer metastasis. Interactions between cancerous and non-cancerous cells can trigger an activation of multiple signaling and gene expression programs that drive disease progression [2,3,4].

Tumor cells can also reprogram immune cells through direct contact or via secreted molecules or soluble factors, leading to pro-oncogenic alterations in signaling, gene expression, and cellular phenotype. These tumor-educated immune cells, including macrophages, dendritic cells, monocytes, platelets, natural killer (NK) cells, and others, can adopt distinct transcription, metabolic, and functional states that can favor tumor survival, invasion and immune evasion [5,6].

Notably, tumor-associated macrophages (TAMs) and myeloid-derived suppressor cells (MDSCs) not only suppress cytotoxic T cell responses but also promote angiogenesis and extracellular matrix (ECM) remodeling, fostering a pro-metastatic niche [7,8,9]. This immune reprogramming can be mediated through sustained exposure to tumor-derived cytokines such as IL6 (interleukin-6), TNFα (tumor necrosis factor-alpha), IL1β (interleukin-1 beta), TGFB (transforming growth factor beta), chemokines, growth factors, and extracellular vesicles [10,11,12,13].

Several studies have highlighted that tumor education extends beyond tissue-resident immune cells, also affecting circulating immune cell populations, which has significant diagnostic and prognostic implications. Hamm et al. demonstrated that tumor-educated circulating peripheral blood monocytes from patients with colorectal cancer (CRC) exhibit distinct transcriptomic signatures, enabling their use as non-invasive biomarkers for diagnosis and disease monitoring [7]. It was also reported that tumor cells can educate NK cells to acquire a metastatic-promoting phenotype, characterized by altered cytokine secretion and reduced cytotoxicity, highlighting the breadth of immune reprogramming across both innate and adaptive compartments [5,6]. Moreover, platelets have emerged as key players in tumor biology. Tumor-educated platelets (TEPs) were shown to undergo RNA profile alterations upon interactions with tumor cells, reflecting the tumor presence and characteristics [14]. Furthermore, TEPs have been implicated in promoting tumor growth and metastasis by transferring tumor-derived RNA and proteins to other cells, thereby influencing the tumor microenvironment and systemic immune response [2,14].

In this study, we aimed to investigate tumor education of immune cells exposed to TNBC cells by analyzing the molecular pathways and functional changes with tumor immune cell interactions under direct or indirect conditions. Here, we describe an in vitro co-culture system using human monocyte cells (THP1) exposed either directly or indirectly to MDA-MB-231 cells. THP1 cells were indirectly exposed via incubation in MDA-MB-231 pre-conditioned media representing TNBC-released soluble factors. THP1 monocytes served as a tractable and widely used model for studying monocyte biology and tumor-induced immune reprogramming, as they share key phenotypic and functional characteristics with primary human monocytes.

We applied transcriptomic and pathway analyses to characterize changes in tumor-educated THP1 monocytes.

Our findings provide an important insight into the mechanism of this transformation and its potential use in diagnostic and therapeutic strategies aimed at detecting, disrupting, or reversing tumor-induced immune reprogramming.

## 2. Materials and Methods

### 2.1. Reagents

Tissue culture media and reagents described below were obtained from Life Technologies, Burlington, ON, Canada. The human leukemic cell lines THP1, BT549, and MDA-MB-231 were obtained from ATCC, Manassas, VA, USA. THP1 cells were maintained in RPMI-1640 (Gibco, Grand Island, NY, USA) supplemented with 10% FBS (Gibco, Grand Island, NY, USA), 1% PenStrep, and 0.05 mM beta-mercaptoethanol (Gibco, Grand Island, NY, USA). MD-MB-231 cells were cultured in DMEM (Gibco, Grand Island, NY, USA) supplemented with 10% FBS (Gibco, Grand Island, NY, USA) and 1% PenStrep (Gibco, Grand Island, NY, USA) and BT-549 cells were cultured in BT549 (DMEM + 10% FBS + 1% PenStrep + 0.01 mg/mL insulin).

### 2.2. Cell Co-Culture Method for RNAseq Preparation

First, adherent cell lines (MDA-MB-231) were seeded into a 6-well (Grenier Bio one, Monroe, NC, USA) plate at 400,000 cells/well. Cells were allowed to attach for 24 h in DMEM (Gibco, Grand Island, NY, USA) supplemented with 10% FBS (Gibco, Grand Island, NY, USA) and 1% PenStrep (Gibco, Grand Island, NY, USA). After 24 h, cells were washed 1× with warm PBS (Milliopore Sigma, Burlington MA, USA), then 400,000 THP1 cells were directly added to each well. THP1 cells were suspended in RPMI-1640 (Gibco, Grand Island, NY, USA) supplemented with 10% FBS (Gibco, Grand Island, NY, USA), 1% PenStrep, and 0.05 mM beta-mercaptoethanol (Gibco, Grand Island, NY, USA). Cells were contacted for 24 h. After contact, THP1 cells were separated from adherent cells. For pre-conditioned cancer media, cancer cells were seeded at 4 million cells in 24 mL THP1. After 24 h, pre-conditioned cancer cell media were centrifuged and filtered through a 0.45 µM filter. THP1 cells were resuspended in THP1 media or pre-conditioned cancer media and cultured for 24 h. Three biological replicates were used for each condition. Both cell fractions were washed once with PBS before RNA extraction. RNA was extracted using a MirVana MiRNA extraction kit (AM1560) (Life Technologies, Burlington, ON, Canada), and eluted in RNAse free water (Invitrogen, Waltham, MA, USA). The RNA purity was checked with a 260/230 ratio, using Take3 on Cytation C10 (Agilent BioTek, Winooski, VT, USA), and the integrity was checked using an Agilent 2100 Bioanalyzer (Agilent, Winooski, VT, USA). RNA with sufficient quality and purity was stored at −80 °C until being sent to Novogene (Sacramento, CA, USA) for library preparation and RNA sequencing.

### 2.3. PBMC Preparation

Four BD vacutainer CPT tubes (BD Biosciences, San Jose, CA, USA), each containing 8 mL of blood, were collected from 4 healthy donors. Sample collection was performed as part of the IDBC clinical study (https://clinicaltrials.gov/study/NCT04495244 (accessed on 5 December 2025)); Health Research Ethics Board of Alberta Cancer Committee approval HREBA.CC-17-0032). Tubes were inverted 8–10 times and centrifuged at 1500–1800 rcf for 20 min at room temperature within 2 h of blood collection. The PBMC layer was removed and donor PBMCs were pooled prior to washing with PBS. Cells were spun in PBS at 300 rcf for 15 min. The supernatant was removed and the cells were resuspended in RPMI GlutaMAX + 10% FBS + 1% PenStrep for co-culture proliferation assays with MDA-MB-231.

### 2.4. Proliferation Assay

MDA-MB-231 (DMEM + 10% FBS + 1% PenStrep), BT549 (DMEM + 10% FBS + 1% PenStrep + 0.01 mg/mL Insulin), or MCF10A (MEBM supplemented with MEGM kit from Lonza) cells were seeded in a 96-well plate (Nunc Millipore Sigma, Burlington MA, USA) at 5000 cell/well and allowed to attach for 24 h. THP1 cells were spun down and resuspended in fresh THP1 media and added to MDA-MB-231, BT-549, or MCF10A cells at a 1:1 ratio. PBMCs were spun down and resuspended in fresh RPMI GlutaMAX + 10% FBS + 1% PenStrep and added to MDA-MB-231 cells at a 2:1 ratio. After 24 h, 20 µL/well of CellTiter 96^®^ Aqueous One Solution Reagent (Promega, Madison, WI, USA) was added to each well and allowed to incubate for 1–3 h until adequate color development was achieved. The absorbance at 490 nm was read on a Cytation C10 (Agilent BioTek, Winooski, VT, USA) plate reader.

For indirect contact, pre-conditioned MDA-MB-231 or BT-549 media were prepared by seeding 4 million MDA-231 or BT-549 cells in 24 mL of RPMI + 10% FBS + 1% PenStrep + 0.05 mM beta-mercaptoethanol (THP1 indirect contact) or in RPMI GlutaMAX + 10% FBS + 1% PenStrep (PBMC indirect contact) overnight. Spent media were harvested by centrifugation at 20,000× *g* for 10 min and filtered through a 0.45 µM filter (Millipore Sigma, Burlington, MA, USA). THP1 or PBMC cells were spun down and resuspended in fresh THP1, fresh PBMC, pre-conditioned MDA-MB-231, or pre-conditioned BT-549 media (THP1 only) and added to MDA-MB-231 or BT549 cells at a 1:1 ratio for THP1 and 2:1 ratio for PBMCs for 24 h prior to the addition of CellTiter 96^®^ Aqueous One Solution Reagent (Promega, Madison, WI, USA).

### 2.5. IL-6 and TGFB ELISA

Cell culture: MDA-MB-231 cells were cultured in DMEM (Life Technologies, Burlington, ON, Canada) + 10% FBS (Life Technologies, Burlington, ON, Canada) + 1% PenStrep (Gibco, Grand Island, NY, USA) on T75 flasks and incubated at 37 °C in a humidified atmosphere enriched with 5% CO_2_. THP1 cells were incubated at 37 °C in a humidified atmosphere enriched with 5% CO_2_. PBMCs were collected from 8 mL of whole blood in BD vacutainer CPT tubes (BD Biosciences, San Jose, CA, USA). Tubes were inverted 8–10× and centrifuged at 1500–1800× rcf for 20 min at room temperature. The PBMC layer was removed, washed with PBS, and centrifuged at 300 rcf for 15 min. The resulting PBMCs were resuspended in RPMI GlutaMAX (Life Technologies, Burlington, ON, Canada) + 10% FBS + 1% PenStrep for contact experiments. MDA-MB-231 cells were plated at 400,000 cell/well in 6-well plates in DMEM media for 24 h. Pre-conditioned MDA-MB-231 media were prepared by seeding 3 million MDA-MB-231 cells in RPMI + 10% FBS + 1% Pen Strep + 0.05 mM beta-mercaptoethanol or RPMI GlutaMAX + 10% FBS + 1% Pen Strep overnight. Pre-conditioned media were harvested as described above. THP1 or PBMC cells were spun down and resuspended in fresh THP1, fresh PBMC, or pre-conditioned MDA-MB-231 media and added to MDA-MB-231 cells at a 1:1 ratio for THP1 cells and 2:1 ratio for PBMCs. After 24 h of contact, cell media were centrifuged at 11,000× *g* for 5 min to remove cellular debris. Secreted IL-6 and TGFB protein was measured by IL-6 ELISA (catalog #KE00139, Proteintech, Rosemont, IL, USA) or TGFB ELISA (catalog #100647, ABCAM, Cambridge, UK) according to the manufacturer’s instructions. The absorbance at 450 nm was read on the Cytation C10 imager (Biotek, Winooski, VT, USA).

### 2.6. RNA Sequencing/Bioinformatics

RNA was harvested as described above and RNA sequencing and analyses were performed by Novogene Corporation (Sacramento, CA, USA). Data were analyzed through the use of IPA (QIAGEN, Inc., Venlo, The Netherlands) https://www.qiagenbioinformatics.com/products/ingenuitypathway-analysis (accessed on 5 December 2025)).

### 2.7. Imaging

THP1 cells were cultured as above, for 24 h in both pre-conditioned and naïve RPMI 1640 media. After incubation, cells were dyed with 5 µg/mL of Hoechst 33342 (ThermoFisher Scientific, Waltham, MA, USA) and 5 µg/mL of CellMask Deep Red (ThermoFisher Scientific, Waltham, MA, USA). Cells were incubated for 10 min at 37 °C, then washed 2× with warm PBS (Milliopore, Sigma, Burlington, MA, USA). After washing, cells were wet-mounted and imaged at 20× using a Cytation C10 (Agilent BioTek, Santa Clara, CA, USA) confocal imager. Phase contrast images were taken at the focal plane, and fluorescent images were taken using a maximum intensity projection of 10 slices, with the step size being determined using Nyquist sampling. All montages were created using a 10% overlap, with the final image being calculated using linear blending.

### 2.8. Statistical Analysis

All experiments were performed with at least three independent biological replicates unless otherwise indicated. Data are reported as the mean ± standard deviation (SD). Statistical analyses were carried out in Microsoft Excel using two-tailed Student’s *t*-tests to compare differences between groups. A *p*-value < 0.05 was considered statistically significant. Exact n values are provided in the corresponding figure legends.

## 3. Results

### 3.1. Human Monocyte Cell Model, THP1, Shows Transcriptional Alteration After Exposure to TNBC Cells

**Figure 1:**
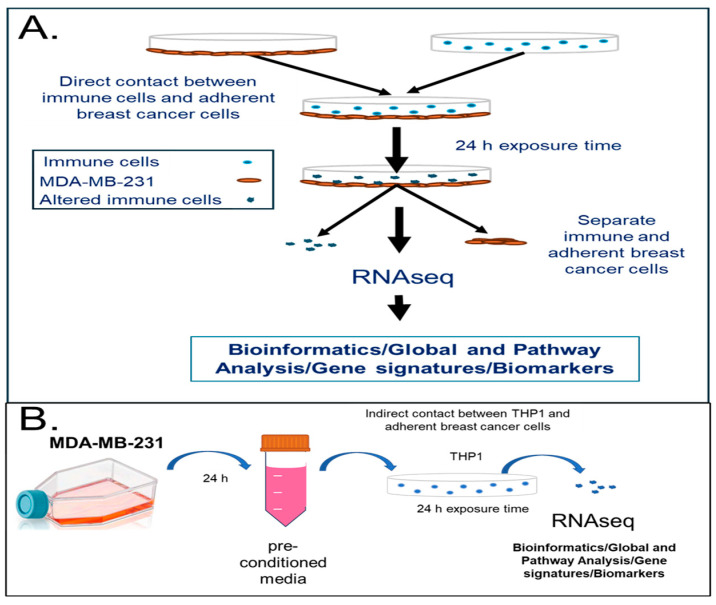
In vitro model system to study transcriptomic changes in human immune cells upon direct or indirect contact with the TNBC cell line, MDA-MB-231. (**A**) Human monocytic cells (THP1) were contacted with MDA-MB-231 TNBC cells for 24 h in RPMI-1640 media in 6-well plates. Cells were separated and RNA analyzed with RNAseq. (**B**) Human monocytic cells (THP1) were incubated with pre-conditioned media from the TNBC cell line (MDA-MB-231) for 24 h. Cells were collected, RNA-isolated, and analyzed using RNA sequencing (RNAseq). A pathway analysis performed using IPA (Qiagen Inc). A minimum of three biological replicates were used for each condition.

Human monocytes can be modulated by cancer cells or secreted factors into carrying pro-oncogenic signals [7,15]. We hypothesized that the intercellular communication between cancer and immune cells can lead to the reprogramming of the immune cells into pro-cancerous or cancerous phenotypes. To investigate the molecular mechanisms behind the transformation of immune cells upon exposure to cancer cells or tumor-cell-secreted factors, we implemented a model of immune–cancer co-culture with TNBC cells (MDA-MB-231) contacted with a human monocytic cell line, THP1, both directly and indirectly. This was followed by RNAseq and pathway analyses (Figure 1). Figure 1A demonstrates the workflow for the direct immune–cancer cell exposure where the adherent MDA-MB-231 cells were plated, allowed to attach, and contacted with THP1 for 24 h. Figure 1B describes the indirect contact in vitro model where the THP1 cells were cultured in the pre-conditioned media from MDA-MB-231 for 24 h. In both cases, THP1 cells were collected after 24 h of exposure and an analysis was performed followed by a differential gene expression analysis using Ingenuity Pathway Analysis (IPA).

**Figure 2:**
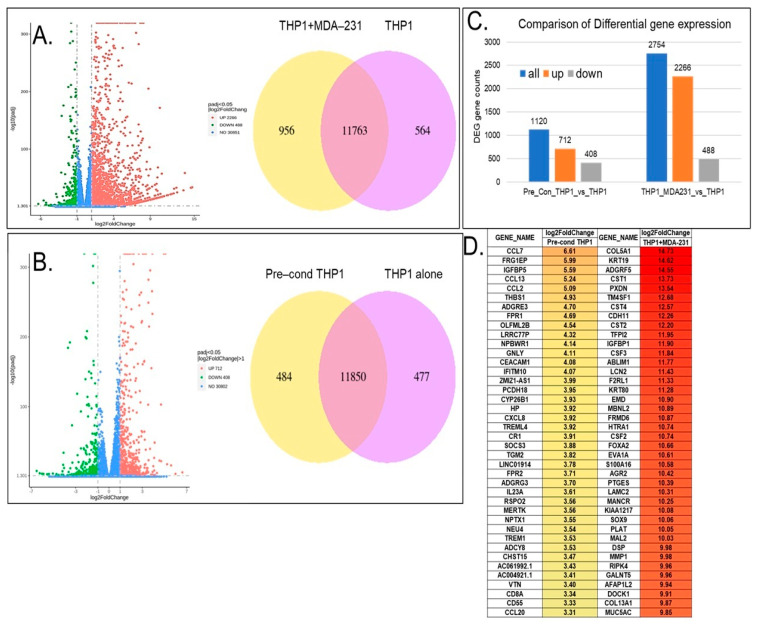
Differential gene expression of THP1 cells contacted with the MDA-MB-231 cell line directly (**A**) or cultured in MDA-MB-231 pre-conditioned media (**B**) for 24 h. (**A**) Volcano plot showing the differentially expressed genes in THP1 cells contacted with MDA-MB-231. Venn diagrams represent shared and unique genes in THP1 cells with or without contact with MDA-MB-231. (**C**) Comparison of the differential gene expression between THP1 cells contacted with MDA-MB-231 indirectly vs. THP1 cells alone and directly vs. THP1 cells alone. (**D**) Top 40 differentially expressed genes upregulated in THP1 cells upon direct contact with MDA-MB-231 or after culturing in MDA-MB-231 pre-conditioned media for 24 h.

Exposure of THP1 cells to both TNBC breast cancer cells or TNBC pre-conditioned media caused major alterations in the THP1 transcriptome. Figure 2A demonstrates that there were over 2700 significant differentially expressed genes (DEGs) (|fold change| > 2, *p* value < 0.05) in THP1 cells after 24 h of direct exposure to MDA-MB-231. Among the differentially expressed genes, 2266 DEGs were significantly upregulated and 488 were downregulated (Figure 2A,C). Indirect THP1 contact in the pre-conditioned media led to significant changes in 1120 genes, of which 712 genes were upregulated and 408 were downregulated (Figure 2B,C). Figure 2D presents a heat map of the top 40 upregulated genes in THP1 upon direct vs. indirect contact with MDA-MB-231. Among the top 40 most upregulated genes in THP1 cells contacted with MDA-MB-231, there were multiple genes involved in cell–cell communication, adhesion, and extracellular modeling, suggesting that indeed immune–breast cancer cell interaction can lead to pro-oncogenic gene expression changes in the THP1 model. For example, *ADGRF5*, a regulator of cell surface signaling, is linked to extracellular matrix interactions and remodeling; *HTRA1* modulates insulin-like growth factor (IGF) signaling and thereby influences growth control; CDH11, a cadherin family member, mediates calcium-dependent cell–cell adhesion and contributes to macrophage-stromal crosstalk; *CST1* has been implicated in breast cancer proliferation and progression. In addition, several markers of epithelial–mesenchymal transition (EMT) were also upregulated (FC > 2), suggesting that contact with breast cancer cells of an aggressive phenotype (e.g., TNBC) can promote EMT in immune cells. Pre-conditioned medium exposure of THP1 showed that the top 40 upregulated genes involved immune and defense responses, chemotaxis, and extracellular-matrix-associated regulators.

**Figure 3:**
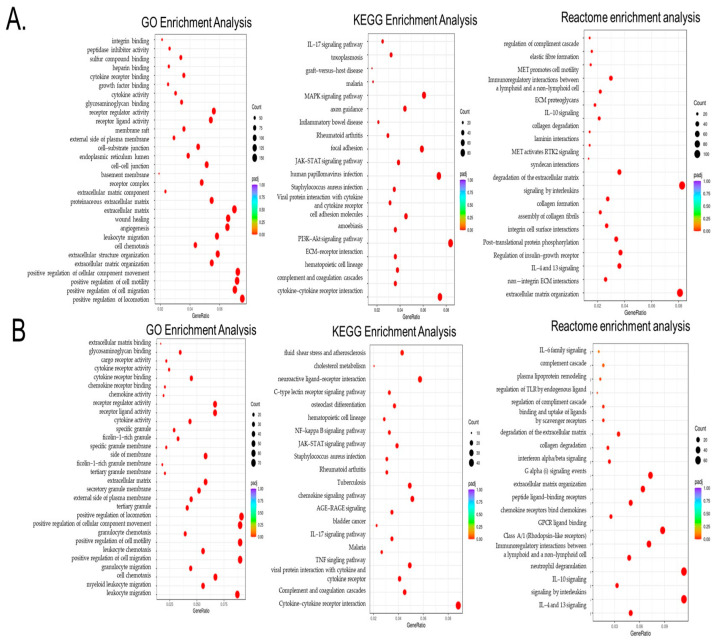
Gene Ontology (GO), KEGG, and Reactome enrichment analyses of the differential genes in THP1 cells contacted with MDA-MB-231 directly (**A**) or via MDA-MB-231 pre-conditioned media indirect THP1 contact. (**B**) In the results of the GO enrichment analysis, the most significant 30 terms were selected for display. KEGG pathways with padj < 0.05 indicate significant enrichment. The most significant 20 Reactome pathways were selected for display. In the figure, the abscissa is the ratio of the number of differential genes linked with the Reactome pathway to the total number of differential genes, and the ordinate is Reactome pathway. The size of a point represents the number of genes annotated to a specific Reactome pathway, and the colors from red to purple represent the significant size of the enrichment. When the results were less than 30, all terms were drawn, as shown in the following figure. In this figure, the abscissa is the GO term. The ordinate is the significance level of GO term enrichment. Higher values correspond to higher significance.

Next, we sought to interrogate the mechanisms behind the observed cancer exposure immune transformations and performed a Gene Ontology (GO) enrichment analysis of significant differential genes in THP1 cells contacted with MDA-MB-231 both directly and through pre-conditioned media with indirect contact. As shown in Figure 3A,B, there were multiple enriched pathways related to gene expression changes associated with positive regulation of cell migration, cell motility, cell chemotaxis, extracellular matrix organization, cell–cell junctions, cytokine receptor binding, leukocyte migration, increased cytokine activity and extra cellular junctions, and growth-factor-related pathways, as well as wound healing and angiogenesis. Among shared processes between direct and indirect TNBC–THP1 interactions were cell chemotaxis, positive regulation of cell motility, cell migration, leukocyte migration, and elevated cytokine and chemokine activity.

To further identify signaling hubs, we performed a KEGG (Kyoto Encyclopedia of Genes and Genomes) enrichment analysis to identify important pathways associated with the differentially expressed genes as compared to the whole genome background. Figure 3A,B demonstrate that the exposure of THP1 to TNBC cells leads to significant enrichment of cytokine–cytokine and ECM receptor interaction, as well as PI3K-AKT, MAPK, and notably JACK-STAT3 signaling pathways, all of which are mechanistically linked to STAT3 activation and immune reprogramming. Among these, IL6/JAK-STAT signaling emerged as one of the most relevant pathways.

Finally, we also evaluated the Reactome enrichment analysis, which similarly revealed enrichment of extracellular matrix organization, integrin cell surface interaction, non-integrin membrane extracellular matrix (ECM) interactions, multiple interleukin-related pathways (such as IL4, IL13, and IL10), and post-translational phosphorylation, among others, which support cell migration and tumor–immune crosstalk.

These results suggest that both direct and indirect TNBC–THP1 cell interaction led to the activation of cell trafficking pathways, as well as upregulation of multiple oncogenic processes associated with progression and metastasis, adaptive immune response, inflammation and immune evasion. These findings strongly suggest that breast cancer cells can reprogram immune cells into a pro-oncogenic state that is capable of promoting cancer dissemination.

### 3.2. Tumor Education Reprograms Immune Cells Towards a Pro-Tumorigenic Phenotype: Pathway Analysis of the Key Regulators Involved in Tumor-Associated Reprogramming of the THP1 Cells Exposed to TNBC Cell Line Directly or Indirectly

To further define the molecular mechanisms associated with tumor-induced immune reprogramming that may drive metastatic progression, we used Ingenuity Pathway Analysis (IPA) to analyze transcriptomic data from our model tumor-educated THP1 cells.

**Figure 4:**
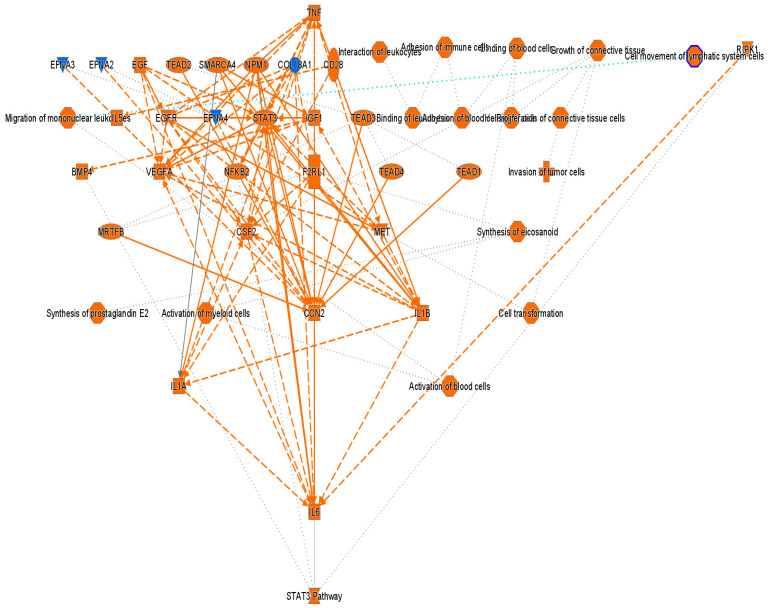
Graphical summary of the Ingenuity Pathway Analysis (IPA) core analysis of differentially expressed genes (|fold change| > 2, *p* value < 0.05) in THP1 cells after 24 h direct exposure to MDA-MB-231. Orange = activation, blue = inhibition. Solid lines indicate direct and dotted lines indicate indirect interactions.

Figure 4 presents a graphical summary of the canonical pathway analysis of direct THP1 cell exposure to MDA-MB-231 cells with the involved upstream regulators. This hierarchical pathway analysis reveals the activation of multiple oncogenic processes including increased proliferation, tumor cell invasion, leukocyte binding, migration, and cell motility, which are collectively consistent with the GO analysis.

**Figure 5:**
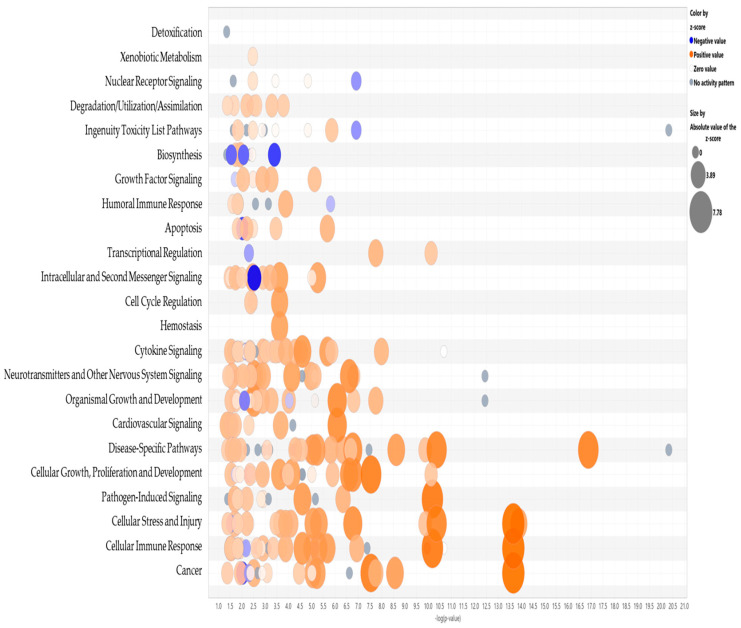
Bubble graph representation of the significant canonical pathways with an activation Z score > 1.5. Blue represents inhibition, orange–activation.

Notably, key upstream regulators such as VEGF, STAT3, IL6/IL1A, TNF, NFκB, and EGFR were significantly upregulated. Among these, cellular communication network factor 2 (CCN2) appeared to play a key role in the observed tumor-induced immune reprogramming. It has been reported to be involved in cell adhesion, proliferation, angiogenesis, and migration in many cell types including fibroblasts, myofibroblasts, and endothelial and epithelial cells [16]. Tumor necrosis factor alpha (TNF) was also identified as a master regulator, acting through multiple points of activation including NFkB, CCN2, VEGFA, EGFR, and STAT3 (Figure 5).

Table 1 summarizes a comparison analysis of canonical pathways significantly altered in THP1 cells following direct or indirect contact (pre-conditioned media) with MDA-MB-231 for 24 h. Only significant pathways with a Z score > 1.5 and *p* value < 0.05 are reported. These results revealed that our model of tumor-educated THP1 cells had widespread reprogramming marked by activation of signaling cascades associated with inflammation, immune cell trafficking, tissue remodeling, and tumor-supportive functions.

The most significantly enriched pathways were identified as the S100 family signaling pathway, pathogen-induced cytokine storm signaling, wound healing signaling, phagosome formation, hepatic fibrosis/hepatic stellate cell activation, and axonal guidance signaling. The activation of these pathways suggests a shift toward a hyper-inflammatory, cytokine-rich phenotype. Enrichment of pro-inflammatory cytokine pathways involving IL6, IL8, IL13, IL17, TNF, and interferons; macrophage polarization; and cytokine storm responses suggests that tumor–immune cell interactions promote a sustained inflammatory microenvironment indicating the establishment of a hyper-inflammatory, cytokine-rich phenotype.

Simultaneous activation of HIF1α, JAK/STAT3, and NFκB pathways further indicates the involvement of transcriptional programs that drive cell survival, angiogenesis, and immune evasion. Enrichment of pathways involved in macrophage classical and alternative activation, such as TREM1 signaling, and cytokine storm responses suggests that model immune cells acquire a mixed activation state supporting both inflammation and immune suppression, i.e., hallmarks of tumor-promoting macrophages.

Upregulation of epithelial–mesenchymal transition (EMT)-related pathways, such as regulation of EMT by growth factors, HIF1α, TGFβ, and wound healing signaling pathways, further suggests a role for immune cells in supporting tumor cell plasticity, invasiveness, and metastasis.

The enrichment of tumor microenvironment remodeling pathways, including macrophage activation, fibrosis signaling, and extravasation/migration pathways (such as leukocyte migration), underscores the potential role of tumor education in creating a pro-metastatic niche. Additional enrichment of pathways such as RIG-1-like receptor signaling, the inflammasome, and micropinocytosis suggests involvement of innate immune sensing and metabolic adaptation. This is consistent with the functional transformation in model cells in response to tumor-derived contact cues.

Taken together, these findings suggest that tumor-educated immune cells, particularly monocytes and macrophages, can undergo functional reprogramming that may support inflammation-driven tumor progression, immune suppression, and metastatic dissemination.

To investigate key regulatory drivers of the transformation described above, we conducted an upstream regulator analysis using IPA. Table 2 lists the top 30 significantly activated regulators, including TNF, NPM1, SMARCA4, RRAS2, IFNG, TGFB1, and STING1. The identified upstream regulators are implicated in cell proliferation, epigenetic remodeling, cell adhesion, cytokine activity, and immune suppression, which are indicative of coordinated reprogramming of immune cells toward a tumor-promoting, pro-inflammatory, and immunosuppressive phenotype.

The upregulation of key mediators such as TNF, IL1A/B, INFG, INFA2, and TGFB1 in tumor-educated immune cells suggests a shift toward a pro-inflammatory yet immunosuppressive phenotype. Simultaneously, the activation of MAPK signaling and survival regulators (PRKCD, PDGF-BB) indicates enhanced immune cell activation and migration. Upregulation of epigenetic and transcriptional regulators such as NPM1, SMARCA4, ASPSCR1-TFE3 and TFEB further suggests that tumor signals drive transcriptional reprogramming of model immune cells, creating a stable pro-oncogenic phenotype. Moreover, the activation of STING1 and interferon alpha suggests a sustained innate immune activation program, which, under chronic stimulation may promote immune exhaustion, tolerance or compensatory suppression of anti-tumor responses [17,18,19].

Collectively, these findings indicate that tumor-educated immune cells in this study underwent a coordinated rewriting of inflammatory and epigenetic pathways that can support a tumor-permissive immune state characterized by chronic inflammation, immune evasion in response to imposed conditions and ultimately, facilitation of metastasis. These changes appear to be driven by the coordinated activity of inflammatory cytokines, innate immune signaling pathways and key transcriptional regulators, revealing multiple potential molecular targets for therapeutic intervention.

### 3.3. Functional Reprogramming of Tumor-Educated Immune Cells Reveals Broad Molecular and Pathological Associations

To further define the biological impact of tumor-induced reprogramming of the immune cells, Ingenuity Pathway Analysis (IPA) was used to generate altered top molecular and cellular conditions, as well as their associations with the pathological conditions. The analysis revealed widespread transcriptional reprogramming across multiple molecular and cellular functions, indicative of a profound phenotypic shift in THP1 cells. The top 4 significantly enriched functional categories shared between model tumor-educated monocytes directly and indirectly exposed to MDA-MB-231 included cellular movement, cell-to-cell signaling and interaction, cellular development, and cellular growth and proliferation. Enhanced cell migration and intercellular communication may facilitate sustained immune–tumor cell interactions, underscoring the role of dynamic immune–tumor cell interactions that can drive the transformation of monocytes toward a pro-oncogenic phenotype.

IPA also revealed strong associations with multiple disease and disorder categories. The shared categories between direct and indirect THP1 cell exposure to TNBC cells included immunological and inflammatory diseases, as well as organismal injury and abnormalities (Table 3). Notably, cancer was predominantly associated with direct THP1–tumor contact exposure, whereas inflammatory responses and infectious disease pathways were more prominent in indirect exposure conditions. These outcomes point to shared molecular programs between tumor-driven immune modulation and immune responses observed in chronic inflammation and infection. Moreover, these findings suggest that tumor-educated immune cells may adopt a gene expression profile resembling a pathogen-exposed or chronically inflamed immune phenotype.

### 3.4. Effects of Tumor-Driven Immune Cell Reprogramming on Proliferation and Cytokine Release

#### 3.4.1. Tumor-Induced Cell Activation Enhances THP1 and PBMC Proliferation Through Direct and Indirect Contact (Via Pre-Conditioned MDA-MB-231 Media) with Breast Cancer Cells

**Figure 6:**
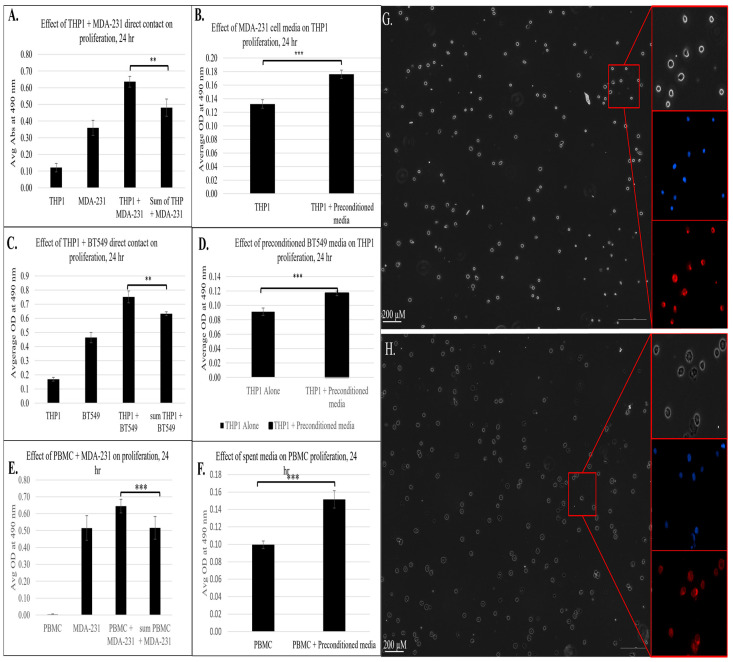
The effect of direct or indirect contact between cancer and immune cells from a phenotypic assay based on proliferation of 3 biological replicates (n = 3); ** = *p* < 0.01, *** = *p* < 0.001, measured using a CellTiter 96 Aqueous kit (Promega). (**A**) Effect of direct contact between THP1 and MDA-MB-231 on proliferation. (**B**) Effect of pre-conditioned MDA-MB-231 media on THP1 proliferation. (**C**) Effect of direct contact between THP1 and BT-549 on immune cell proliferation. (**D**) Effect of pre-conditioned BT-549 media on proliferation of THP1 cells. Effect of direct (**E**) and indirect (pre-conditioned media) (**F**) contact between human PBMCs and MDA-MB-231 on immune cell proliferation. Sum of THP-1 + MDA-MB-231 refers to the combined proliferation measurements from the individual cell lines. (**G**) Morphology of THP1 cells after 24 h of incubation in regular (**G**) or MDA-MB-231 pre-conditioned media (**H**). Phase contrast images. Right panels are magnified views of corresponding areas in red boxes. Nuclei stained with Hoechst33342 shown in blue and cell membrane stained with CellMask Deep Red in red.

Next, we sought to validate the transcriptomic and pathway analysis findings in our model in vitro cell system. As the IPA, GO, and KEGG enrichment analyses revealed that the contact between immune and cancer cells led to the activation of multiple pathways associated with cellular growth, proliferation, and migration, we investigated whether the immune–cancer cell communication results in functional changes in cell growth. To assess this, we adapted the co-culture system to a 96-well format and measured cell proliferation using a CellTiter 96 Aqueous kit (Promega), with and without cell contact, after 24 h of incubation. As shown in Figure 6A, the co-culture of THP1 monocytes with MDA-MB-231 breast cancer cells for 24 h led to a synergistic increase in proliferation relative to monocultures of the cell type. This finding functionally confirms the transcriptomic data, suggesting that the immune–cancer cell model triggers immune cell reprogramming toward a proliferative phenotype that may contribute to metastatic progression.

To determine whether soluble factors alone could induce these effects, we evaluated THP1 proliferation following exposure to MDA-MB-231 conditioned media. As shown in Figure 6B, indirect exposure of THP1 cells to breast cancer resulted in a significant increase in cell proliferation, indicating that the tumor-derived soluble mediators were sufficient to trigger an immune cell response consistent with RNAseq and IPA results. Similar results were observed with another TNBC cell line, BT-549, where both direct contact (Figure 6C) and exposure to pre-conditioned media (Figure 6D) led to a significant increase in the proliferation of THP1 cells. These findings indicate that TNBC-driven immune reprogramming occurs across multiple TNBC models. Importantly, co-culture with non-tumorigenic epithelial MCF10a cells did not increase THP1 proliferation (Figure S1), confirming that this proliferative effect is TNBC-specific rather than a generic epithelial phenomenon.

In parallel with the transcriptional and functional changes, we evaluated morphological changes to THP1 cells upon exposure to pre-conditioned media for 24 h (Figure 6G,H). In the control condition (panel 6G), THP1 cells retained a mostly rounded morphology with centrally located nuclei (blue) and limited cytoplasmic spread (red), which is typical of undifferentiated or M0-like monocytes. In contrast, THP1 cells cultured in pre-conditioned media (panel 6H) displayed irregular outlines with signs of partial spreading and more prominent cytoplasmic extensions, while the nuclei remained intact and evenly distributed. These changes are consistent with a shift toward cytoskeletal remodeling and differentiation toward an activated macrophage-like state. These staining patterns further support the interpretation that tumor-derived factors can reprogram monocytes away from an M0-like morphology toward an activated, polarized phenotype.

To investigate the key findings from our model system in a physiologically relevant context, we isolated peripheral blood mononuclear cells (PBMCs) from healthy donors and co-cultured them with MDA-MB-231 breast cancer cells under both direct and indirect contact conditions. This approach allowed us to investigate whether interactions with cancer cells elicited a similar pro-proliferative response in primary immune cells. As shown in Figure 6E, PBMCs directly co-cultured with MDA-MB-231 cells exhibited a significant increase in proliferation compared to both cell cultures alone. This finding aligns with the prior THP1 observation and confirms that immune–tumor cell crosstalk can trigger proliferative programs consistent with immune programming. To further evaluate the role of tumor-derived soluble factors, we cultured PBMCs in MDA-MB-231 pre-conditioned media. Notably, these PBMCs also demonstrated a significant increase in proliferation (Figure 6F), indicating that tumor-cell-secreted factors alone could drive immune cell activation. This finding underscores the potential role of tumor-secreted factors in driving immune cell reprogramming and has an important implication in tumor metastasis. Together, these results corroborate the proliferative THP1 phenotype and have significance in a more relevant immune context, showing that tumor–immune cell communication, whether via direct or indirect contact, can promote immune cell proliferation and transformation into a pro-oncogenic state contributing to immune suppression, metastasis, and tumor-supportive niche formation.

#### 3.4.2. Cytokine Induction and STAT3 Pathway Activation Increases in Tumor-Educated Immune Cells Through Direct and Indirect Contact with Breast Cancer Cells

The pathway analysis using IPA revealed activation of multiple inflammatory and oncogenic networks in tumor-exposed THP1 cells (Figure 3, Table 1). This analysis revealed strong activation of cytokine- and inflammation-associated signaling pathways, including IL-6, IL-8, IL-13, and IL-17 signaling of NF-κB and JAK/STAT-related cascades, alongside broader immune and inflammatory processes such as inflammasome and interferon signaling, pathogen-induced cytokine storm signaling, macrophage activation, and hypercytokinemia pathways. Based on these findings, we specifically examined cytokine-related gene expression changes to identify mediators likely driving the observed transcriptional reprogramming of THP1 cells. This analysis highlighted the consistent induction of pro-inflammatory cytokines such as *IL-1β, IL-6, IL-8, IL-10, IL-17A/F*, and *TNF*, which are known to play central roles in immune activation, macrophage polarization, and tumor–immune cell crosstalk (Table 4). The upregulation of canonical STAT3 target genes, including *IL6*, *IL10, IL11, OSM, LIF, SOCS3, CISH, BCL2*, and *CCND1,* strongly suggests activation of STAT3 signaling in THP1 cells exposed to TNBCs. These transcriptional changes are consistent with cytokine-driven STAT3 activation and reflect a shift toward survival, proliferation, and immune-modulatory phenotypes, even in the absence of direct protein-level validation.

The analysis of activation and antigen presentation markers supported this transcriptomic reprogramming. Direct contact led to strong upregulation of *CD14* (8.53-fold), *ICAM* (9.61-fold), *CD163* (3.81-fold), *HLA-DRA* (9.19-fold) and *HLA-DPB1* (6.62-fold). THP1 cells exposed to pre-conditioned media retained *CD14*, *CD86*, *CD163*, and *ICAM1* induction with significant changes in *HLA-DRA* or *HLA-DPB1*. This indicates that soluble tumor factors can be sufficient for partial activation and adequate to drive immune cell polarization and functional reprogramming.

To investigate the predicted activation of inflammatory cytokine signaling pathways revealed by our IPA and transcriptomic analyses, we assessed the protein expression levels of IL6, a central pro-inflammatory cytokine and a top-ranked regulator identified in the IPA analysis. IL6 plays a key role in tumor–immune cell communication, immune cell polarization, and the establishment of a chronic inflammatory microenvironment facilitating metastasis.

**Figure 7:**
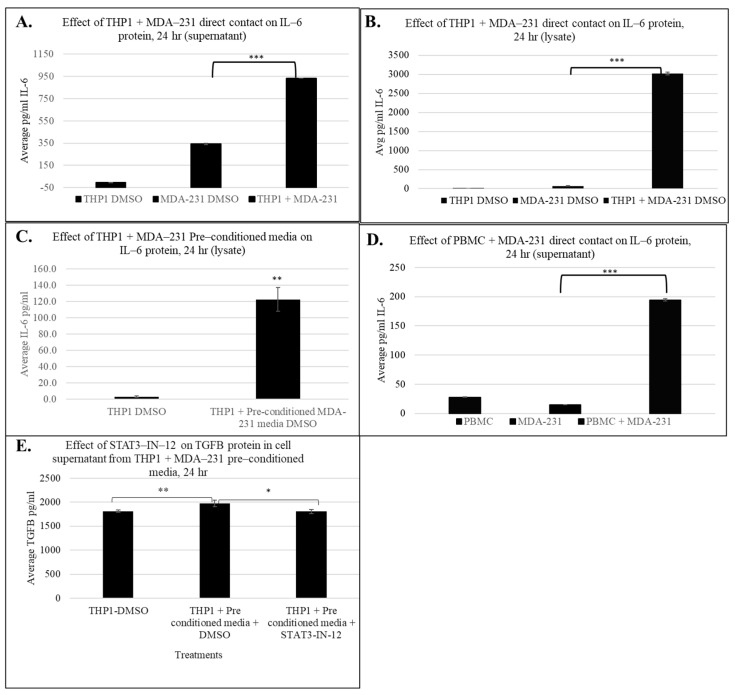
Effect of immune cell + MDA-231 contact on IL-6 and TGFB protein levels in cell supernatant and lysate after 24 h. IL6 ELISA measuring IL6 in the THP1 supernatant (**A**) and lysate (**B**) with direct MDA-231 contact and in the cell lysate with pre-conditioned MDA-231 media (**C**). Induction of IL-6 protein with PBMC + MDA-231 contact in cell supernatant (**D**). TGFB protein expression with THP1 + MDA-231 pre-conditioned media and 5 uM STAT3-IN-12 treatment for 24 h (**E**). The shown data represent the mean ± SD of three biological replicates with *p* values as follows: * = *p* < 0.05, ** = *p* < 0.01, *** = *p* < 0.001.

Using ELISA, we measured the IL6 protein levels in THP1 cell lysates and supernatants post MDA-MB-231 24 h exposure. Figure 7A demonstrates that direct contact between THP1 and MDA-MB-231 cells for 24 h resulted in a significant increase in secreted IL6 protein levels compared to individual monocultures (~935 fold vs. THP1 and 2.7-fold vs. MDA-MB-231 alone). Figure 7B demonstrates a striking induction (~3000-fold increase) of intracellular IL6 protein levels in cell lysates in co-cultured THP1 cells, further confirming activation of IL6 expression in response to tumor–immune cell communication.

To determine whether soluble factors from tumor cells could also induce IL6 production in immune cells, we exposed THP1 cells to MDA-MB-231 conditioned media for 24 h and measured IL6 protein levels. As shown in Figure 7C, indirect exposure also significantly elevated IL6 protein levels in cell lysates, consistent with the IPA results described above, suggesting that both soluble mediator and physical cell–cell interactions can contribute to immune cell activation.

Finally, to evaluate the relevance of these findings in a physiologically relevant immune cell type, we performed IL6 measurements using freshly isolated PBMCs. Consistent with the THP1 data, PBMCs directly co-cultured with MDA-MB-231 cells showed a robust increase in IL6 secretion (~7-fold) into the supernatant (Figure 7D), indicating that tumor-induced immune education occurs in primary human cells. These results are in strong agreement with IPA and transcriptomics data suggesting that IL6 signaling is a key inflammatory axis activated during tumor–immune cell interaction.

To further investigate cytokines implicated in STAT3-associated immune reprogramming, we assessed IL-10 and TGFβ at the protein level. While IL-10 was identified at the transcriptomic level, it was not detectable under the experimental conditions, suggesting that its regulation may require additional stimuli. Conversely, TGFβ secretion was significantly increased in THP1 cells exposed to pre-conditioned MDA-MB-231 media. Importantly, TGFβ is a well-established mediator of myeloid immune suppression and tumor-promoting inflammation and has been shown to cooperate with STAT3 signaling to reinforce pro-tumorigenic immune phenotypes [20,21,22]. Notably, this increase in TGFβ secretion was counteracted by pharmacological inhibition of STAT3 using STAT3-IN-12, indicating that tumor-conditioned media induce TGFβ production in a STAT3-dependent manner. These results highlight a broader cytokine network influenced by STAT3 activity, reinforcing its potential as a therapeutic target in TNBC-driven immune reprogramming.

Together, these transcriptional, morphological, and marker expression data suggest that TNBC-driven tumor education of immune cells promotes a spectrum of monocyte reprogramming involving activation of potent pro-inflammatory programs including cytokine signaling, which are likely contributing to immune cell reprograming, tumor progression, and the creation of a metastasis-promoting microenvironment.

#### 3.4.3. Small-Molecule Inhibitors Suppress Tumor-Induced Immune Cell Proliferation

**Figure 8:**
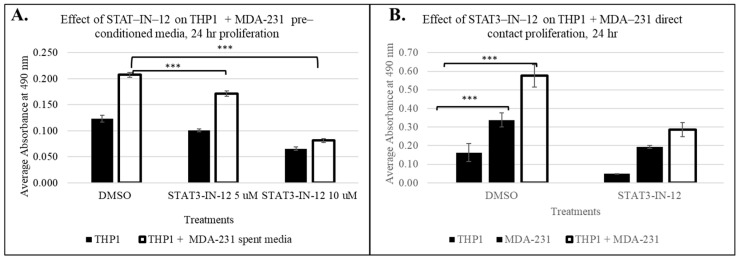
The effect of the small-molecule STAT3 inhibitor STAT3-IN-12 on breast-cancer-induced THP1 proliferation in pre-conditioned MDA-MB-231 media, n = 3 at 24 h The shown data represent the mean ± SD of three biological replicates with *p* values as follows: *** = *p* < 0.001 (**A**). (**B**) Direct contact of THP1 with MDA-MB-231. STAT-IN-12 treatments in (**B**) represent the mean ± SD of two biological replicates, measured using CellTiter 96 Aqueous kit (Promega).

Next, we sought to assess whether this tumor-induced immune cell reprogramming can be reversed by small-molecule therapeutics. As the pathway analysis identified the significant activation of the STAT3 signaling axis following cancer-driven immune education, we investigated whether STAT3 inhibition could reverse or suppress the functional consequences of this reprogramming. Using our direct and indirect contact models, THP1 cells were exposed to TNBC cells or conditioned media with or without STAT3-IN-12 treatment, a selective STAT3 inhibitor (concentration and treatment time determined from a dose curve, Supplemental Figure S2). As shown in Figure 8, STAT3-IN-12 significantly reduced the proliferation of THP1 induced by both direct and indirect contact with MDA-MB-231 cells. These findings indicate that STAT3 activation is a critical driver of tumor-induced proliferation, and its inhibition can mitigate this effect.

## 4. Discussion

Crosstalk between cancer and non-cancer cells within the tumor microenvironment can occur through direct contact or secreted molecules and facilitate the transformation of normal cell function into a pro-oncogenic phenotype that may lead to cancer progression and metastasis. Our data demonstrate that TNBC cells (MDA-MB-231 and BT549) can reprogram THP1 monocytes and primary human PBMCs into a proliferative, cytokine-activated state via both contact-dependent and soluble-factor-mediated mechanisms. The lack of proliferation with MCF10a co-culture confirms that these effects are cancer-specific. THP1 monocytes provide as a tractable reductionist model [15,23,24] to define core tumor-driven immune reprogramming pathways, and importantly the consistency between THP1 and primary PMBCs phenotypic and cytokine responses supports the translational relevance of these findings.

Such tumor education of immune cells can lead to dynamic changes in immune cell signaling, gene expression, function, or phenotype and has the potential to be utilized for the creation and establishment of new cancer detection and treatment approaches. Therefore, we aimed to elucidate molecular mechanisms of immune–cancer cell communication to further utilize these findings in biomarker and drug discovery strategies.

In this study, we aimed to investigate the molecular and functional consequences of tumor-induced immune education using co-culture in vitro cell models. By exposing THP1 monocytes or human PBMCs to TNBC cell lines through either direct or indirect contact, we demonstrated that immune cells undergo significant phenotypic and transcriptomic changes, including increased proliferation and widespread activation of multiple inflammatory and oncogenic signaling pathways, including upregulation of inflammatory mediators such as IL6 and TGFβ. These findings suggest that tumor–immune cell communication may be sufficient to induce a reprogrammed, pro-tumorigenic immune phenotype that can influence malignancy and immune evasion.

Notably, the pathway enrichment analysis revealed strong activation of signaling cascades involved in cytokine signaling, immune activation, cellular stress, and injury responses. These included STAT3/IL6, IL8, IL17, NFkB, INFG, and p38 MPAK signaling cascades, consistent with a transition toward a chronic, pro-inflammatory, and immunosuppressive state [25]. In parallel, canonical pathways associated with EMT, cell migration, and survival were also significantly upregulated, consistent with previous studies demonstrating that tumors can exploit inflammatory cytokines and transcriptional regulators to convert immune cells into facilitators of tumor growth and progression [13,26]. STAT3 pathway activation in THP1 cells is well supported through transcriptomic data, including upregulation of canonical STAT3 targets (SOCS3, CISH, BCL3, JUNB, PIM1) and increased expression of STAT3-activating ligands (IL6, IL11, LIF, OSM) in response to TNBC contact.

Multiple lines of transcriptomic evidence in tumor-educated THP1 cells point to STAT3 pathway activation, including upregulation of canonical STAT3 targets (SOCS3, CISH, BCL3, JUNB, PIM1) and increased expression of STAT3-activating ligands (IL6, IL11, LIF, OSM) in response to TNBC contact. This STAT3-centirc program is well-aligned with established mechanisms underlying myeloid-derived suppressor cell (MDSC) expansion and tumor-associated macrophage (TAM) polarization [27]. The phenotype observed here shares key features with early MDSC-like states, including enhanced cytokine signaling, proliferative capacity, and transcriptional programs associated with immune suppression rather than effective anti-tumor immunity [28,29,30].

In addition to these transcriptomic changes, tumor-educated THP1 cells displayed distinct morphological alterations consistent with a transition toward an activated macrophage-like phenotype, aligned with early M1/M2 polarization states. The morphological shift provides additional evidence that tumor-educated monocytes can simultaneously support inflammatory signaling and immunosuppression, which may be sufficient to drive immune cell differentiation and functional reprogramming.

Functionally, this reprogramming was evident through increased proliferation of both THP1 cells and PBMCs following tumor cell exposure, in both direct and indirect co-culture models. Interestingly, these results suggest that tumor-secreted factors are sufficient to elicit proliferation and potentially immunosuppressive phenotypes in circulating monocytes. Elevation of IL6 expression levels observed in THP1 cells and PBMCs exposed to breast cancer cells further supports the involvement of cytokine-mediated signaling in this reprogramming process. IL6 is known to promote STAT3 activation, which has been widely implicated in cancer-induced immune tolerance and tumor progression [31,32].

Importantly, these findings have potential implications for resistance to current immunotherapies. STAT3-driven myeloid reprogramming is known to impair antigen presentation, suppress effector cell function, and promote the exclusion of dysfunction of cytotoxic lymphocytes within tumors. Such mechanisms have been directly linked to reduced responsiveness to immune checkpoint inhibitors including anti-PD-1 and anti-CTLA-4 therapies. The tumor-educated immune phenotype described here may, therefore, contribute to both intrinsic and acquired resistance to checkpoint blockade by establishing a systemic, cytokine-driven immunosuppressive environment that limits effective anti-tumor immune activation [33,34].

To investigate whether induced changes could be reversed through pharmacological intervention, we tested the STAT3 inhibitor, STAT3-IN-12, in both co-culture settings. STAT3 inhibition led to significantly reduced proliferation of tumor-educated THP1 cells, emphasizing the critical role of STAT3 signaling in mediating cancer-induced immune activation. Importantly, these results support the concept that STAT3-targeted therapies have the potential to disrupt the transcriptional programs underlying immune cell transformation in the tumor microenvironment. By blocking key transcriptional programs responsible for immune cell reprogramming, STAT3 inhibition can suppress or reverse the pro-tumorgenic phenotype and can potentially restore immune surveillance capacity, thereby complementing existing tumor-targeted therapies and potentially enhancing the responsiveness to checkpoint blockade.

Overall, our data reveal that tumor education initiates a coordinated rewriting of inflammatory and epigenetic programs in monocytes and macrophages. This results in a tumor-permissive immune state characterized by chronic inflammation, immune suppression, and enhanced metastatic potential. This transformation is mediated by cytokine signaling and transcriptional regulation, identifying multiple potential molecular targets for therapeutic intervention.

Importantly, the results of this study support the hypothesis that tumor–immune cell crosstalk extends beyond the tumor microenvironment. Circulating immune cells such as monocytes, NK cells, or platelets can retain disease-induced transcriptomic and functional signatures, offering the opportunity for new methods of cancer detection or monitoring and new methods of treatment [4,5,6,35]. Together these results support a new paradigm in cancer therapy through blocking tumor-driven immune education with small-molecule inhibitors, with the potential to enhance the treatment efficacy and broaden the utility of immune-modifying agents in oncology.

## 5. Conclusions

In conclusion, our findings demonstrate that cancer cells can alter immune signaling and differentiation mechanisms to reprogram immune cells into a pro-tumor immunomodulatory phenotype. This transformation is not only linked to cancer-specific pathways but also reflects broader immunopathological signatures, underscoring the therapeutic potential for targeting tumor-educated immune cells for both biomarker development and immunotherapeutic strategies. Among the key regulators, STAT3 signaling emerged as a central mediator of this reprogramming and was effectively inhibited by small-molecule intervention, reversing aspects of the tumor-educated phenotype. These findings support a new shift in cancer therapy, advocating for strategies that target not only the tumor cells but also corrupted immune components of the tumor microenvironment. Such strategies may lead to improved therapeutic efficacy, help overcome resistance mechanisms, and broaden the clinical utility of immune-modulating agents in oncology.

## Figures and Tables

**Table 1:** Ingenuity Pathway Analysis (IPA) results for the significant pathways affected upon 24 h of THP1 contact with MDA-MB-231. Only significant pathways are presented with *p* values < 0.05.

Canonical Pathways	THP1 + MDA-231 Z Score	Pre_Cond THP1 Z Score
S100 Family Signaling Pathway	7.78	3.76
Phagosome Formation	7.31	3.83
FAK Signaling	7.04	3.46
Pathogen-Induced Cytokine Storm Signaling Pathway	6.88	4.90
Pulmonary Fibrosis Idiopathic Signaling Pathway	6.74	4.04
Cardiac Hypertrophy Signaling (Enhanced)	6.22	2.92
Hepatic Fibrosis Signaling Pathway	6.10	2.34
IL-17 Signaling	5.52	3.96
Role Of Osteoclasts In Rheumatoid Arthritis Signaling Pathway	5.42	4.12
Tumor Microenvironment Pathway	5.40	3.27
Macrophage Classical Activation Signaling Pathway	5.38	2.98
Breast Cancer Regulation by Stathmin1	5.29	2.95
Pulmonary Healing Signaling Pathway	5.25	2.83
Wound Healing Signaling Pathway	5.09	2.27
Role Of Chondrocytes In Rheumatoid Arthritis Signaling Pathway	5.05	3.80
Colorectal Cancer Metastasis Signaling	4.84	2.99
IL-8 Signaling	4.77	2.99
Neuroinflammation Signaling Pathway	4.33	2.45
HIF1α Signaling	4.32	2.60
MSP-RON Signaling In Cancer Cell Pathway	4.20	2.71
Role of Hypercytokinemia/Hyperchemokinemia in the Pathogenesis of Influenza	4.20	2.67
ID1 Signaling Pathway	4.14	2.13
Macrophage Alternative Activation Signaling Pathway	4.13	2.65
Heparan Sulfate Biosynthesis (Late Stages)	3.00	2.65
Heparan Sulfate Biosynthesis	3.00	2.65
Multiple Sclerosis Signaling Pathway	4.13	1.79
HMGB1 Signaling	4.08	2.36
Role of JAK Family Kinases in IL-6-type Cytokine Signaling	4.04	2.11
NOD1/2 Signaling Pathway	3.78	2.18
RAC Signaling	3.46	2.45
Regulation Of The Epithelial Mesenchymal Transition By Growth Factors Pathway	3.66	2.67
TREM1 Signaling	3.64	3.21
Role of IL-17F in Allergic Inflammatory Airway Diseases	3.46	2.83
Dermatan Sulfate Biosynthesis	3.46	2.45
Osteoarthritis Pathway	3.40	2.68
ERBB Signaling	3.36	2.33
Pyroptosis Signaling Pathway	3.30	2.32
Dermatan Sulfate Biosynthesis (Late Stages)	3.16	2.45
Chondroitin Sulfate Biosynthesis (Late Stages)	3.16	2.45
Chondroitin Sulfate Biosynthesis	3.16	2.45
Pancreatic Adenocarcinoma Signaling	3.15	2.31
Role Of Osteoblasts In Rheumatoid Arthritis Signaling Pathway	3.11	3.00
Differential Regulation of Cytokine Production in Intestinal Epithelial Cells by IL-17A and IL-17F	3.00	2.00
IL-13 Signaling Pathway	2.86	2.31
HOTAIR Regulatory Pathway	2.86	1.81
Glioma Invasiveness Signaling	2.83	3.00
Role of IL-17A in Psoriasis	2.83	2.65
Oncostatin M Signaling	2.83	1.63
Macropinocytosis Signaling	2.45	2.00
IL-6 Signaling	2.71	2.07
p38 MAPK Signaling	2.50	1.51
Systemic Lupus Erythematosus In B Cell Signaling Pathway	4.14	1.63
IL-17A Signaling in Gastric Cells	2.45	2.00
Inflammasome Pathway	2.45	1.63
Interferon Signaling	2.33	2.45
Natural Killer Cell Signaling	2.27	2.83
Inhibition of Angiogenesis by TSP1	2.12	1.63
Th2 Pathway	1.94	2.33
Leukocyte Extravasation Signaling	1.67	2.00
LXR/RXR Activation	−1.83	−1.71
Superpathway of Cholesterol Biosynthesis	−3.16	−2.00

**Table 2:** Upstream regulators activated in THP1 cells after 24 h of exposure to MDA-MB-231. Only significant pathways with a Z score > 1.5 and *p* value < 0.05 are reported.

Upstream Regulators	THP1 + MDA-231 Z Score	Pre-Cond THP1 Z Score
TNF	9.35	6.19
NPM1	8.32	5.90
SMARCA4	7.80	3.21
RRAS2	7.68	4.38
IFNG	6.84	4.02
TGFB1	6.72	4.13
STING1	6.66	5.28
NFkB (complex)	6.59	4.20
PDGF BB	6.44	4.54
IL1A	6.41	3.77
CG	6.03	4.21
ERK	5.81	4.36
RIGI	5.74	3.35
MAVS	5.68	3.72
TGM2	5.66	5.16
ASPSCR1-TFE3	5.65	3.32
NONO	5.60	3.93
P38 MAPK	5.58	3.54
IFNA2	5.58	3.08
SYVN1	5.56	3.00
STAT1	5.50	2.00
PRKCD	5.45	3.08
RELA	5.43	3.45
Interferon alpha	5.40	3.62
IL1B	5.36	4.35
STAT3	5.35	3.95
TFEB	5.28	3.83
SORL1	5.25	1.73
RIPK2	5.21	4.26
IL-1R	5.16	3.35

**Table 3:** IPA results of THP1 cells exposed to MDA-231 for 24 h directly or indirectly, top molecular and cellular conditions (**A**), and diseases and biofunctions (**B**).

(A) Top Molecular and Cellular Conditions	THP1 + MDA-231		Pre-Cond THP1	
	*p* Value Range	#Molecules	*p* Value Range	# Molecules
Cellular movement	1.47 × 10^−11^–4.41 × 10^−64^	647	1.19 × 10^−7^- 1.07 × 10^−28^	273
Cell-to-cell signaling and interaction	6.88 × 10^−12^–1.61 × 10^−36^	476	9.39 × 10^−8^- 3.77 × 10^−22^	244
Cell death and survival	1.49 × 10^−11^ 1.77 × 10^−30^	590	-	-
Cellular development	1.47 × 10^−11^–8.79 × 10^−27^	664	1.17 × 10^−7^–3.06 × 10^−14^	275
Cellular growth and proliferation	1.47 × 10^−11^–8.79 × 10^−27^	663	1.49 × 10^−11^–1.77 × 10^−30^	272
Cellular function and maintenance	-	-	1.17 × 10^−7^–3.06 × 10^−14^	184
**(B)** Top Diseases and Disorders	THP1 + MDA-231		Pre-cond THP1	
	*p* value range	# Molecules	*p* value range	# Molecules
Immunological disease	1.55 × 10^−11^–3.90 × 10^−50^	1083	6.38 × 10^−8^–7.16 × 10^−39^	409
Inflammatory disease	7.78 × 10^−12^–3.90 × 10^−50^	569	1.12 × 10^−7^–7.16 × 10^−39^	280
Organismal injury and abnormalities	1.55 × 10^−11^–3.90 × 10^−47^	2208	1.18 × 10^−7^–7.16 × 10^−39^	886
Cancer	1.55 × 10^−11^–6.26 × 10^−47^	2193	-	-
Dermatological disease and conditions	8.17 × 10^−12^–1.73 × 10^−40^	1713	-	-
Immunological disease	1.55 × 10^−11^–3.90 × 19^−50^	1083	-	-
Inflammatory response	-	-	4.02 × 10^−8^–1.51 × 10^−23^	298
Infectious disease	-	-	4.02 × 10^−9^–1.08 × 10^−22^	218

**Table 4:** Gene expression changes of pro-inflammatory cytokines and chemokines, STAT3-related genes, and antigen presentation or macrophage activation markers upon direct and indirect contact of THP1 with MDA-MB-231.

Gene Name	Direct Contact (THP1+ MDA-MB-231)		Indirect Contact (Pre-Cond THP1)	
	Fold Change	Padj	Fold Change	Padj
Pro-inflammatory cytokines and family members				
*IL6*	175.66	1.3 × 10^−43^	ns	ns
*IL1B*	7.78	1.4 × 10^−266^	9.47	0.0
*IL1A*	31.61	4.4 × 10^−16^	4.91	1.8 × 10^−2^
*IL7*	5.96	6.1 × 10^−3^	ns	ns
*IL15*	7.66	3.9 × 10^−6^	ns	ns
*IL23A*	6.51	1.9 × 10^−86^	12.23	1.5 × 10^−215^
*IL32*	4.70	1.2 × 10^−38^	ns	ns
*IL27*	ns	ns	4.01	8.1 × 10^−4^
*Anti-inflammatory/immunosuppressive cytokines*				
*IL10*	4.91	7.5 × 10^−4^	ns	ns
*TGFB1*	ns	ns	2.58	0.0
*TGFB2*	4.40	8.6 × 10^−34^	ns	ns
*STAT3-related ligands often activating JAK/STAT3*				
*LIF*	132.29	1.9 × 10^−47^	3.99	3.7 × 10^−2^
*OSM*	7.19	2.4 × 10^−56^	7.65	1.1 × 10^−65^
*IL11*	24.44	6.7 × 10^−14^	ns	ns
*IL6*	175.66	1.3 × 10^−43^	ns	ns
*IL10*	4.91	7.5 × 10^−4^	ns	ns
*IL27*	3.22	9.6 × 10^−3^	4.01	8.1 × 10^−4^
*IL23A*	6.51	1.9 × 10^−86^	12.23	1.5 × 10^−215^
*SOCS3*	19.61	0.0	14.69	3.4 × 10^−266^
*CISH*	3.01	2.3 × 10^−55^	3.20	2.3 × 10^−67^
*CXCL1*	9.68	1.4 × 10^−187^	8.99	3.5 × 10^−165^
*CXCL2*	11.73	4.1 × 10^−45^	4.32	3.6 × 10^−10^
*CXCL3*	13.46	8.4 × 10^−21^	5.26	3.7 × 10^−6^
*CXCL8*	9.09	0.0	9.09	0.0
*CCND1*	13.84	0.0	ns	ns
*BCL2L1*	3.31	1.4 × 10^−122^	ns	ns
*BCL2*	-2.33	1.4 × 10^−191^	ns	ns
*PIM1*	6.14	0.0	2.41	2.5 × 10^−60^
*JUNB*	2.50	3.6 × 10^−135^	2.06	2.4 × 10^−88^
*SAA1*	67.23	4.0 × 10^−216^	ns	ns
*SAA2*	400.95	2.2 × 10^−23^	ns	ns
*VEGFA*	2.64	1.4 × 10^−155^	2.02	1.9 × 10^−80^
*HP*	6.21	5.4 × 10^−76^	15.10	8.7 × 10^−234^
*CD163*	3.81	2.2 × 10^−27^	2.18	2.2 × 10^−7^
*HLA-DRA*	9.19	8.7 × 10^−259^	ns	ns
*BCL3*	4.84	0.0	2.51	1.4 × 10^−112^
*Chemokines (monocyte and neutrophil recruitment)*				
*CXCL1*	9.68	1.4 × 10^−187^	8.99	3.5 × 10^−165^
*CXCL2*	11.73	4.1 × 10^−45^	4.32	3.6 × 10^−10^
*CXCL3*	13.46	8.4 × 10^−21^	5.26	3.7 × 10^−6^
*CXCL6*	2.72	4.5 × 10^−4^	3.87	3.9 × 10^−7^
*CXCL8*	9.09	0.0	9.09	0.0
*CXCL11*	6.13	1.4 × 10^−16^	ns	ns
*CXCL13*	2.68	2.5 × 10^−2^	4.07	3.0 × 10^−4^
*CXCL16*	2.93	1.2 × 10^−10^	2.23	7.5 × 10^−6^
*CCL4*	2.42	6.6 × 10^−3^	ns	ns
*CCL7*	123.41	1.7 × 10^−7^	97.53	1.1 × 10^−6^
*CCL8*	14.17	5.1 × 10^−27^	5.70	6.8 × 10^−8^
*CCL13*	26.02	9.6 × 10^−14^	37.68	2.1 × 10^−18^
*CCL20*	3.22	3.7 × 10^−6^	9.93	3.1 × 10^−39^
*CCL2*	ns	ns	34.15	0.0
*CCL8*	ns	ns	5.70	6.8 × 10^−8^
*CCL24*	ns	ns	4.87	9.0 × 10^−7^
*Colony-stimulating factors and related growth factors*				
*CSF1*	57.44	0.0	ns	ns
*CSF3*	3673.88	2.1 × 10^−22^	ns	ns
*CSF2*	1709.15	2.2 × 10^−18^	ns	ns
*VEGFA*	2.64	1.4 × 10^−155^	2.02	1.9 × 10^−80^
*Acute phase/STAT3-responsive secreted factors*				
*SAA1*	67.23	4.0 × 10^−216^	ns	ns
*SAA2*	400.95	2.2 × 10^−23^	ns	ns
*Cytokine receptors and signaling components*				
*IL10RA*	2.29	9.3 × 10^−91^	2.89	6.8 × 10^−194^
*TGFBR2*	3.33	6.8 × 10^−184^	2.89	6.8 × 10^−194^
*JAK3*	9.91	1.3 × 10^−18^	3.24	1.8 × 10^−3^
*Antigen presentation/macrophage activation markers (functional)*				
*CD14*	8.53	0.0	8.51	0.0
*CD86*	ns	ns	2.24	1.5 × 10^−2^
*ICAM1*	9.61	0.0	2.43	1.3 × 10^−78^
*HLA-DRA*	9.19	8.7 × 10^−259^	ns	ns
*HLA-DPB1*	6.62	4.5 × 10^−61^	ns	ns
*CD163*	3.81	2.2 × 10^−27^	2.18	2.2 × 10^−7^

ns = not significant.

## Data Availability

The raw data supporting the conclusions of this article will be made available by the authors, without undue reservation.

## Supplementary Materials

The following supporting information can be downloaded at: https://www.mdpi.com/article/10.3390/cancers18010116/s1, Figure S1: The effect of direct contact between non-tumorigenic epithelial MCF10a and immune cells (THP1) from a phenotypic assay based on proliferation n = 3; measured using CellTiter 96 Aqueous kit (Promega).; Figure S2: The effect of small molecule STAT3 inhibitor, STAT3-IN-12 on breast cancer induced THP1 proliferation through indirect contact with MDA-MB-231 measured using CellTiter 96 Aqueous kit (Promega), at 24, 46 and 72 h.

## Abbreviations

The following abbreviations are used in this manuscript:

TNBC	Triple negative breast cancer
TME	Tumor microenvironment
NK	Natural killer cells
TAMs	Tumor-associated macrophages
MDSCs	Myeloid-derived suppressor cells
ECM	Extracellular matrix remodeling
TEPs	Tumor-educated platelets
CRC	Colorectal cancer
DEGs	Differentially expressed genes
IGFs	Insulin-like growth factors
EMT	Epithelial–mesenchymal transition
GO	Gene Ontology
CCN2	Cellular communication network factor 2
TNF	Tumor necrosis factor alpha
PBMCs	Peripheral blood mononuclear cells
