# Supplementary material for: Tumor–Immune Cell Crosstalk Drives Immune Cell Reprogramming Towards a Pro-Tumor Proliferative State Involving STAT3 Activation
Source: Cancers (Basel). 2025 Dec 30;18(1):116. doi: 10.3390/cancers18010116 (PMC12784678; doi:10.3390/cancers18010116)
Supplement: Supplementary file 1 [file cancers-18-00116-s001.zip › cancers-4057568-supplementary.pdf]

Supplementary materials

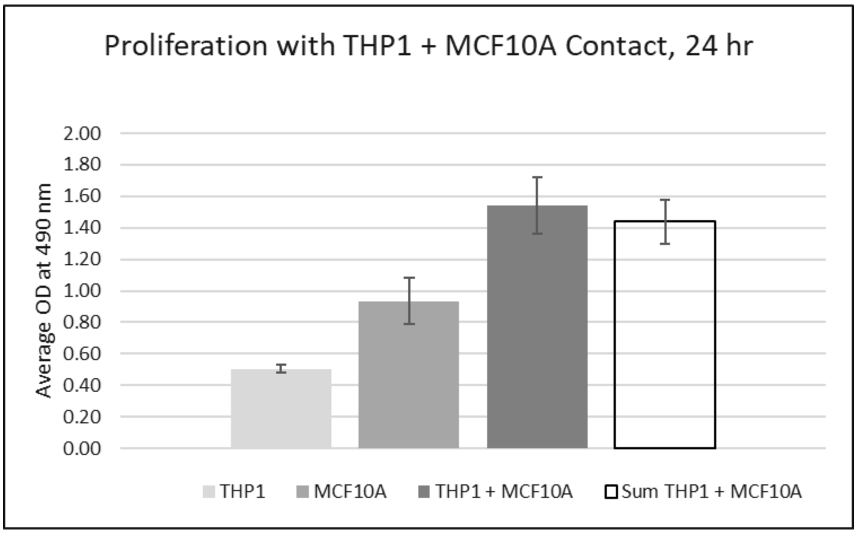

Figure S1. The effect of direct between non-tumorigenic epithelial MCF10a and immune cells (THP1) from a phenotypic assay based on proliferation n=3; **=p<0.01, ***=p<0.001 measured using CellTiter 96 Aqueous kit (Promega).

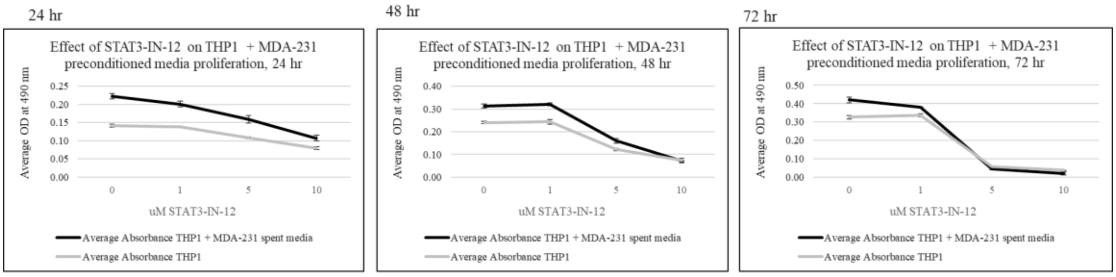

STAT3-IN-12 uM	Average Absorbance THP1 + MDA-231 spent media 24 hr	Stdev	Average Absorbance THP1 24 hr	Stdev
0	0.223	0.01	0.142	0.004
1	0.201	0.01	0.138	0.002
5	0.159	0.01	0.108	0.003
10	0.107	0.01	0.081	0.004

STAT3-IN-12 uM	Average Absorbance THP1 + MDA-231 spent media 48 hr	Stdev	Average Absorbance THP1 48 hr	Stdev
0	0.313	0.008	0.240	0.01
1	0.321	0.006	0.244	0.01
5	0.160	0.010	0.122	0.004
10	0.071	0.002	0.074	0.01

STAT3-IN-12 uM .	Average Absorbance THP1 + MDA-231 spent media 72 hr	Stdev	Average Absorbance THP1 72 hr	Stdev
0	0.420	0.015	0.326	0.01
1	0.380	0.003	0.338	0.01
5	0.046	0.007	0.058	0.001
10	0.020	0.009	0.037	0.001

Figure S2. The effect of small molecule STAT3 inhibitor, STAT3-IN-12 on breast cancer induced THP1 proliferation through indirect contact with MDA-MB-231 measured using CellTiter 96 Aqueous kit (Promega), at 24, 46 and 72 h.
